# Economic burden of beta-thalassemia/Hb E and beta-thalassemia major in Thai children

**DOI:** 10.1186/1756-0500-3-29

**Published:** 2010-01-30

**Authors:** Arthorn Riewpaiboon, Issarang Nuchprayoon, Kitti Torcharus, Kaemthong Indaratna, Montarat Thavorncharoensap, Bang-on Ubol

**Affiliations:** 1 Department of Pharmacy, Faculty of Pharmacy, Mahidol University, Bangkok, Thailand; 2 Department of Pediatrics, Faculty of Medicine, Chulalongkorn University, Bangkok, Thailand; 3 Department of Pediatrics, Phramongkutklao College of Medicine, Bangkok, Thailand; 4 Faculty of Economics, Chulalongkorn University, Bangkok, Thailand; 5 Department of Pediatrics, Saraburi Hospital, Saraburi, Thailand

## Abstract

**Background:**

Hemoglobin E beta-thalassemia (β-thalassemia/Hb E) has a variable severity, and the cost of treatment has not been well studied. The aim of this study was to analyze the societal cost of caring for children with β-thalassemias in Thailand. The study was designed as a prevalence-based cost-of-illness analysis in a societal perspective. Medical records from three public hospitals of children aged 2-18 years with β-thalassemia/Hb E and homozygous β-thalassemia were reviewed for direct medical cost determination. For direct non-medical cost and indirect cost, a family member was interviewed.

**Findings:**

It was found that 201 patients with β-thalassemia/Hb E (91%) and homozygous β-thalassemia (9%) were recruited for this study. Ninety-two (46%) were severe thalassemia and 109 (54%) were mild to moderate severity. The annual average cost of treatment was US$950; 59% was direct medical cost, 17% direct non-medical cost, and 24% indirect cost. The costs were differentiated by some potential predictors. Significant predictor variables were: hospital, health insurance scheme, blood transfusion pattern, and iron chelation drug use.

**Conclusions:**

The average annual cost per patient was calculated, and the cost model was estimated. These would be applied for national planning, economic evaluation of treatment and prevention interventions, and budget impact analysis.

## Background

Beta-thalassemia (β-thalassemia) is a common inherited blood disorder worldwide. Hemoglobin E/β-thalassemia is particularly common in Southeast Asia and the Indian subcontinent, as well as coastal North America, where Southeast Asian immigrants constitute a substantial percentage of the population [1-3]. With a population of 65 million, 7% with a β-thalassemia trait and 17% with an HbE trait, an estimated 35,000 patients are living with β-thalassemia syndrome in Thailand [4]. Hb E/β-thalassemia has a variable severity [5-7].

Studies on the economic burden of β-thalassemia have been reported from the United Kingdom [8], Canada [9], Israel, Taiwan, Sri Lanka [10], and Myanmar [11]. In Thailand, a study on the lifetime cost of homozygous β-thalassemia in children was conducted in 2001, and estimated the direct medical cost of treatment to be 6,660,000 Thai baht, or approximately US$149,899, over a period of 30 years [12]. This cost estimate was based on expert opinion, covering mainly blood transfusion and iron chelation drugs. There is also a study on the cost-benefits of prevention measures [13]. Based on our knowledge, we have not found any cost-of-illness study of thalassemia employing patient-specific cost data covering both direct and indirect costs in Thailand. We therefore conducted a comprehensive study of the cost of Hb E/β-thalassemia.

## Methods

### Cost-of-illness study design

This study was a prevalence-based cost-of-illness analysis [14]. The prevalence-based approach measures the economic burden of all patients caused by a disease in a given period, usually for one year. This study was analyzed from a societal perspective, the broadest one which includes all costs incurred by all members of society, including the public and private sectors [15]. Please see the supplement file for the detail.

### Study hospitals

In Thailand, public health facilities are major players in the health delivery system, accounting for 80.5% of total inpatient beds. For the public sector, there are 15 teaching hospitals, 63 military hospitals, 25 regional hospitals, 70 general hospitals, and 723 community hospitals (statistics from 2004). The study hospitals were selected on the basis of data accessibility and cooperation (personal relationship among researchers). Study sites were Saraburi Hospital (SB), Phramongkutklao Hospital (PK), and Chulalongkorn Hospital (CL). SB is a 680-bed public regional hospital 108 km north-east of Bangkok. PK is a 1,200-bed military medical college, and CL is a 1,268-bed teaching hospital, both located in Bangkok and under the Thalassemia Registration Project. The inpatient wards' occupancy rates were 92% (SB), 75% (PK), and 82% (CL). This study was approved by the ethics committees of all study hospitals.

### Patients and data collection

Thalassemia is defined by haemoglobin typing. The study group was composed of children aged 2-18 years with β-thalassemia/Hb E and thalassemia major during the fiscal year 2005 (1 October 2004 to 30 September 2005). In this study, patients were classified as "severe" if they met at least one of the following criteria: 1) patients whose onset of anemia was before 2 years of age, and whose first transfusion occurred before 4 years of age; 2) patients who were diagnosed with homozygous β-thalassemia; and 3) patients whose pre-transfusion Hb level was less than 7 g/dL [7,16]. Type of transfusion was classified by frequency of blood transfusion: high (more than 12 times/year); low (6-12 times/year); and occasional (1-5 times/year). Thalassemia complications include symptoms resulting from thalassemia (e.g., congestive heart failure due to anemia or ascending cholangitis). The sample size required in the study was calculated using the formula by Altman [17]. According to the formula, the sample size should not be smaller than ten times the total number of independent variables. Because the number of potential predictor variables in this study is 14 covering age, hospital, health insurance scheme, type of disease, blood transfusion pattern (none, low, occasional, or high), splenectomy, severity, iron chelation use, and complications - the sample size required is 140. Medical records were reviewed for direct medical cost determination. For direct non-medical cost and indirect cost, a family member was interviewed.

### Statistical and sensitivity analysis

Descriptive statistics were used to summarize the dependent and independent variables. Stepwise multiple regression analysis [18] was employed to analyze the relationship between the cost (dependent variable) and potential predictor variables (independent variables). Please see the supplement file for the detail. (see Additional file 1)

## Results

### Demographic and clinical characteristics

Two hundred and one patients were included in the study. The interview response rate was 100%. The compositions by hospitals were: 36% from Saraburi Hospital, 43% from Chulalongkorn Hospital, and 21% from Phramongkutklao Hospital. Males slightly outnumbered females in all hospitals except Saraburi. The patients' average age was 9.52 years (SD = 3.82), and more than half were registered under the Universal Health Coverage Scheme. The major type of the disease was β-thalassemia/Hb E (91%). Approximately 30% had undergone a splenectomy. Around 80% received blood transfusions during the study year. On the other hand, 61% did not receive any iron chelation drug. Eighteen patients (9%) had complications. The complications were: gallstones (2 cases), heart failure (2 cases), autoimmune hemolytic anemia (2 cases) and anti-Hepatitis B (anti Hbs) positive (12 cases). Details are shown in Table 1.

**Table 1:** Patients' characteristics

	Hospital			
	
	Saraburi	Phramongkutklao	Chulalongkorn	Total
Number	72 (35.8%)	42 (20.9%)	87 (43.3%)	201 (100%)
Age; mean years (SD)	9.24 (3.89)	11.38 (3.84)	8.86 (3.48)	9.52 (3.82)
Gender (N = 201)				
Male	34 (47.2%)	24 (57.1%)	49 (56.3%)	107 (53.2%)
Female	38 (52.8%)	18 (42.9%)	38 (43.7%)	94 (46.8%)
Type of payment (N = 201)				
Universal Coverage Scheme (UC)	66 (91.7%)	24 (57.1%)	33 (37.9%)	123 (61.2%)
Civil Servant Medical Benefit Scheme (CSMBS)	1 (1.4%)	12 (28.6%)	11 (12.6%)	24 (11.9%)
Out of pocket	5 (6.9%)	6 (14.3%)	43 (49.4%)	54 (26.9%)
Disease type (N = 201)				
Beta-thal/Hb E	68 (94.4%)	37 (88.1%)	78 (89.7%)	183 (91.0%)
Homozygous beta-thalassemia	4 (5.6%)	5 (11.9%)	9 (10.3%)	18 (9.0%)
Severity (N = 200)				
Severe	38 (52.8%)	14 (34.1%)	42 (48.3%)	94 (47.0%)
Non-severe	34 (47.2%)	27 (65.9%)	45 (51.7%)	106 (53.0%)
Splenectomy* (N = 201)				
Yes	22 (30.6%)	19 (45.2%)	22 (25.3%)	63 (31.3%)
No	50 (69.4%)	23 (54.8%)	65 (74.7%)	138 (68.7%)
Type of blood transfusion (N = 198)				
No	23 (31.9%)	8 (19.0%)	10 (11.9%)	41 (20.7%)
Occasional (1-5 times/year)	24 (33.3%)	1 (2.4%)	15 (17.9%)	40 (20.2%)
Low (6-12 times/year)	24 (33.3%)	22 (52.4%)	33 (39.3%)	79 (39.9%)
High (> 12 times/year)	1 (1.4%)	11 (26.2%)	26 (31.0%)	38 (19.2%)
Iron chelation (N = 201)				
Desferrioxamine	3 (4.2%)	27 (64.3%)	48 (55.2%)	78 (38.8%)
No drug	69 (95.8%)	15(35.7%)	39 (44.8%)	123 (61.2%)
Ferritin level (N = 127)				
≤ 2,500 ng/ml	16 (80.0%)	20 (58.8%)	41 (56.2%)	77 (60.6%)
> 2,500 ng/ml	4 (20.0%)	14 (41.2%)	32 (43.8%)	50 (39.4%)
Complications (N = 201)				
Yes	5 (6.9%)	13 (31.0%)	-	18 (9.0%)
No	67 (93.1%)	29 (69.0%)	87 (100.0%)	183 (91.0%)

* prevalence

### Resource utilization and cost

As reported in Table 2, the patients received treatment as outpatients nearly every month (9 times a year), with 11% being hospitalized. Blood transfusion was performed at an average of 7.4 times per year. The iron chelation drug used was desferrioxamine (by injection, 500 mg vial). Average use was 44 vials per year. However, resource utilization was quite different between hospitals and by disease type. The number of outpatient visits was in the range of 7-11 and 9-13 times a year for different hospitals and different diagnoses, respectively. The number of blood transfusions and hospitalizations was also different. Patients at the regional hospital (Saraburi) received less treatment than those at the other two hospitals located in Bangkok for most medical services except for rate of hospitalization. Severe patients received more treatment than non-severe patients. Blood transfusions and iron chelation drugs used by severe patients were approximately two times more than those by non-severe patients. Similarly, patients with complications consumed more resources than those without complications.

**Table 2:** Average service utilization classified by type of services, patient characteristics, and hospitals

	Outpatient	Hospitalization	Average length of stay (days, SD)		Blood	
					
Category	service (visits, SD)	(%)	Per all patients	Per hospitalized patients	transfusion (times, SD)	Desferrioxamine (vials, SD)
Hospital						
Saraburi (N = 72)	6.74 (3.31)	15 (20.8%)	0.68 (1.76)	3.27 (2.58)	3.69 (3.59)	3.43 (20.42)
Phramongkutklao (N = 42)	9.76 (4.44)	-	-	-	8.64 (5.74)	93.95 (105.41)
Chulalongkorn (N = 87)	11.05 (4.37)	7 (8.0%)	0.69 (3.48)	8.57 (9.74)	9.86 (5.44)	53.77 (82.50)
Disease type						
Beta-thal/Hb E (N = 183)	8.86 (4.31)	20 (10.9%)	0.57 (2.64)	5.25 (6.39)	6.94 (5.57)	39.59 (79.17)
Homozygous beta-thal (N = 18)	13.06 (4.30)	2 (11.1%)	0.22 (0.73)	2.00 (1.41)	12.06 (4.32)	90.33 (83.78)
Severity						
Severe (N = 94)	10.41 (4.14)	16 (17.0%)	0.95 (3.53)	5.56 (7.07)	9.35 (4.93)	58.63 (89.75)
Nonsevere (N = 106)	8.26 (4.46)	6 (5.7%)	0.19 (0.92)	3.33 (2.25)	5.74 (5.72)	31.70 (70.03)
Ferritin level						
≤ 2,500 ng/ml (N = 77)	10.60 (3.51)	5 (6.5%)	0.17 (0.80)	2.60 (2.07)	8.96 (4.55)	51.38 (88.92)
> 2,500 ng/ml (N = 50)	11.76 (3.91)	5 (10.0%)	1.20 (4.60)	12.00 (9.82)	11.24 (4.94)	96.02 (92.76)
Complications						
Yes (N = 18)	11.83 (3.92)	3 (16.7%)	0.56 (1.54)	3.33 (2.52)	10.39 (4.84)	113.44 (123.39)
No (N = 183)	8.98 (4.44)	19 (10.4%)	0.54 (2.61)	5.21 (6.56)	7.10 (5.65)	37.32 (72.18)

Total	201	22 (10.9%)	201	22	201	201
Mean	9.23	n/a	0.54	4.95	7.40	44.13
Standard deviation (SD)	4.46	n/a	2.53	6.16	5.65	80.69
Median	9.00	n/a	0.00	3.00	8.00	0.00
95% CI						
Lower	8.61	n/a	0.19	2.22	6.61	32.91
Upper	9.85	n/a	0.89	7.69	8.18	55.36

Regarding costing, the numbers of individual medical resources were multiplied by their unit costs. The unit costs for frequently used drugs and laboratory investigations are presented in Table 3. The costs were converted from Thai baht to US dollars at the rate of 40.22 baht per US$1 (2005 prices) [19]. Costing results are presented in Table 4. Direct medical cost was separated into cost incurred at the study hospitals, and other health facilities including drug stores and alternative medicine. For cost at the study hospitals, the iron-chelating drug (DFO) was shown separately from other drugs. Routine service referred to cost of visits at outpatient departments and hospital stays in wards. Each item of cost and total cost were presented in the form of subgroup analysis regarding hospital, disease type, severity, ferritin level, and complications. For total cost from a societal perspective, the average cost of patients at the teaching hospital (Chulalongkorn) was US$1,297, three times more than that of the public hospital (Saraburi). Homozygous β-thalassemia had the highest cost (US$1,434). Patients with severe conditions and complications had higher costs than those with non-severe conditions and no complications. In terms of cost composition, iron chelation drugs accounted for 39%, followed by blood transfusion at 21% of the direct medical cost at the study hospitals. In sum, the cost of the disease was composed of 59% direct medical cost, 17% direct non-medical cost, and 24% indirect cost. Please see the supplement files for the detail of sensitivity analysis and cost function. (see Additional file 1)

**Table 3:** Unit cost of drugs and medical services* (US$ at 2005 prices)

	per 100 units	per unit
Aspirin, enteric-coated tablet, 1 grain	0.47	n/a
Desferrioxamine injection, 500 mg	452.26	n/a
Deferasirox tablet, 250 mg	1243	n/a
Folic acid tablet, 5 mg	0.22	n/a
Furosemide injection, 20 mg/2 ml	11.64	n/a
Furosemide tablet, 40 mg	0.67	n/a
Multivitamin syrup, 60 ml	25.48	n/a
Multivitamin tablet	0.52	n/a
Penicillin V tablet, 250 mg	1.86	n/a
LPRC	n/a	13.67
Pre-storage filtered PRC	n/a	11.93
NAT LPRC	n/a	21.13
NAT Prestorage filtered PRC	n/a	19.39
Complete blood count (CBC)	n/a	1.49
Cross-matching	n/a	1.99
Ferritin	n/a	7.71
Hematocrit (Hct)	n/a	0.75
Hemoglobin (Hb)	n/a	2.49
Hemoglobin typing	n/a	6.71

LPRC = leukocyte-reduced packed red cell

Prestorage filtered PRC = leukocyte-depleted packed red cell (LDPRC)

NAT LPRC = nucleic acid-tested LPRC

* From the prices of services of health facilities under the Ministry of Public Health [25].

**Table 4:** Average cost classified by cost composition, patient characteristics, and hospitals (US$ at 2005 prices)

Category	Direct medical cost										Direct	Indirect	Total
				
	Study site								Other	Total	non	Cost	
						
	DFO	Other drugs	Medical material	Blood transfusion	Investigation	Routine service*	Operation	Total	Facilities		medical cost		
Hospital													
Saraburi (N = 72)	15.52	6.53	3.60	50.99	49.99	53.27	12.95	192.84	32.06	224.90	56.81	98.39	380.09
Phramongkutklao (N = 42)	424.91	12.38	25.97	134.91	104.11	59.78	-	762.05	20.65	782.70	181.97	241.56	1206.23
Chulalongkorn (N = 87)	245.18	12.28	79.02	139.42	85.19	91.14	-	650.23	85.96	736.19	228.77	332.42	1297.38
Disease type													
Beta-thal/Hb E (N = 183)	179.05	9.53	39.59	99.18	73.33	69.35	4.01	474.04	57.78	531.82	146.09	224.19	902.10
Homozygous beta-thal (N = 18)	408.54	17.45	54.44	184.25	109.14	87.96	11.05	872.82	4.42	877.24	272.37	284.66	1434.27
Severity													
Severe (N = 94)	265.15	14.13	41.35	136.11	92.12	94.98	7.80	651.63	23.32	674.95	155.53	192.53	1023.01
Nonsevere (N = 106)	143.36	6.89	40.92	81.81	63.42	50.42	1.88	388.70	76.45	465.15	158.97	264.65	888.77
Ferritin level													
≤ 2,500 ng/ml (N = 77)	232.36	8.72	51.65	130.50	91.76	68.83	2.58	586.40	58.41	644.81	200.01	301.38	1146.20
> 2,500 ng/ml (N = 50)	434.26	19.63	73.00	158.19	110.76	114.85	-	910.68	6.10	916.78	222.36	257.55	1396.69
Complications													
Yes (N = 18)	513.07	17.83	32.59	151.57	120.23	88.86	11.05	935.18	7.25	942.43	185.54	257.87	1385.84
No (N = 183)	168.77	9.50	41.74	102.39	72.24	69.27	4008.00	467.91	57.50	525.41	154.63	226.82	906.86

Total													
Mean	199.60	10.24	40.92	106.80	76.53	71.02	4.64	509.75	53.00	562.76	157.39	229.60	949.75
Standard Deviation (SD)	364.92	14.78	72.76	83.33	50.83	95.36	33.69	547.04	247.80	606.10	201.84	520.36	n/a
Median	0.00	3.56	12.99	109.40	77.08	63.65	0.00	323.71	0.00	362.66	92.99	67.81	654.19
95%CI													
Lower	148.85	8.19	30.80	95.21	69.46	57.76	-0.05	433.67	18.54	478.46	129.32	157.23	806.48
Upper	250.36	12.30	51.04	118.39	83.60	84.28	9.33	585.84	87.47	647.06	185.47	301.98	1093.03
% (subtotal)	39.16	2.01	8.03	20.95	15.01	13.93	0.91	100.00					
% (total)										59.25	16.57	24.17	100.00

*Cost of routine service covers cost of outpatient visits and inpatient services. These costs include consultation fee and overhead cost.

(see Additional file 2)

## Discussion

Unlike thalassemia in European countries, a majority of Thai thalassemia patients represented in this study are of intermediate severity. Most children with severe thalassemia in Thailand are under-treated by international standards [16], as evidenced by pre-transfusion hemoglobin below 7 g/dL. Few received iron chelation, and all but very few patients received adequate iron chelation. The choices of high or low transfusion are individually determined by the treating physician, as well as through compliance of the patient's family. There are differences in the practice of thalassemia treatment. While the proportion of types and severity of thalassemia in children were comparable among the three study sites, one-quarter of patients treated in the medical school received high transfusion, > 40% had high ferritin, and more than half received iron chelation. In contrast, all but one patient at Saraburi received high transfusion, only 20% had high ferritin, and < 5% received iron chelation. It is therefore not surprising that costs are significantly higher at medical centers in Bangkok.

Although this study may have limitations on the number of hospitals included, they do represent three major health service settings: namely, public hospitals, teaching hospitals, and military hospitals. In regard to the representativeness of the patients, based on the proportion of newborn thalassemia (thal): β-thal/Hb E, homozygous β-thal, and Hb H disease constitute 30%, 6%, and 64%, respectively. Life spans of β-thal/Hb E, homozygous β-thal, and Hb H disease patients are estimated as 30 years, 10 years, and 60 years, respectively [20,21]. The proportion of β-thal/Hb E to homozygous β-thal was 5:1. By comparison, proportions of β-thal/Hb E and homozygous β-thal in this study are 91% and 9%, respectively (Table 1). The proportion of β-thal/Hb E and homozygous β-thal was thus 10:1. Therefore, the mean cost of the samples may not represent that of the population. In addition, we conducted a subgroup analysis. A weighted average technique can be applied to calculate the mean cost of the population [22]. Other studies used cost at charge or incomplete cost items [11,23,24]. The cost composition included in this analysis is the most complete, since it covers material cost, labor cost and capital cost, including the cost of patient care and non-patient care activities [25]. However, it is a prevalence-based cost-of-illness study, and therefore does not show the whole picture as an incidence-based or lifetime cost of illness, as do other studies. The lifetime cost of thalassemia is presented elsewhere [8,9].

Direct medical cost was approximately 60% of the total cost. Among the direct medical costs, iron chelation drugs accounted for 39%. Iron chelation contributes significantly to the cost of treatment. Adequate iron chelation has been shown to reduce the complications related to thalassemia and iron overload, and reduce the cost of treatment for these complications [26]. Proportion of indirect cost would increase if we can capture productivity loss due to a decrease of working capacity. In addition, it would be higher among adult patients who are all in working age with higher number of death. Please see the supplement files for the detail of sensitivity analysis and cost function. (see Additional file 2)

## Conclusions

In conclusion, to our knowledge, this is the first time that a complete cost-of-illness study of thalassemia has been performed in Thailand. The results provided the cost of taking care of child patients in a societal perspective, covering direct medical cost, direct non-medical cost, and indirect cost. The average annual cost per patient was US$950. Direct medical cost accounted for 59% of the total cost. The costs were classified by hospital type, thalassemia type, severity, and complication. The effects of blood type and iron chelation drug as cost drivers were quantitatively explored. A cost model was fitted; hospital, health insurance, blood transfusion level, iron chelation drug use, and health insurance were significant predictor variables.

## Competing interests

The authors declare that they have no competing interests.

## Authors' contributions

KT, IN, and BU are haematologists. AR and KI are health economists. MT is a pharmacoepidemiologist. All researchers contributed to the study design, supervision of data collection, and reading of the analysis results. AR performed the analyses and wrote the manuscript, and is therefore the first author. All the authors approved the final manuscript.

## Supplementary Material

Additional file 1 **Additional methods, results and discussion**. More detail on costing methods, sensitivity analysis and cost function. Click here for file

Additional file 2 **Table S1-S4**. Results of sensitivity analysis and cost function. Click here for file
