# Supplementary material for: Economic burden of beta-thalassemia/Hb E and beta-thalassemia major in Thai children
Source: BMC Res Notes. 2010 Jan 30;3:29. doi: 10.1186/1756-0500-3-29 (PMC2835719; doi:10.1186/1756-0500-3-29)
Supplement: Additional file 1 — Additional methods, results and discussion. More detail on costing methods, sensitivity analysis and cost function. [file 1756-0500-3-29-S1.DOC]

Additional file 1: More detail on Methods, Results and Discussion

Methods

**Cost-of-illness study design**

Cost of illness is composed of direct medical cost, direct non-medical cost, and indirect cost [1]. Direct medical cost is healthcare-related costs directly spent for the prevention, detection, treatment, continuing care, rehabilitation, and terminal care of patients. This study covers only treatment cost incurred at the study hospitals and additional treatment at other health facilities, including alternative medicines. Direct medical cost is calculated by multiplying the quantity of medical services consumed by their unit costs. The unit cost of medical services used in this study was based on the prices of services of health facilities under the Thailand Ministry of Public Health [2]. The unit prices cover material, labor, and capital costs. Unit costs of drugs are the average purchasing prices at public hospitals [3].

Direct non-medical costs are non-medical costs directly related to medical procedures, such as transportation, meals, accommodations, home modifications, and informal care (care by relatives).

Indirect costs are defined as productivity lost due to the illness. There are two forms of indirect costs: morbidity and mortality costs. Morbidity costs include the value of production losses for those who are sick/absent, unemployed, or restricted from working due to an illness. Mortality costs are calculated as the present value of the loss of production due to premature death caused by illness [1]. In this study, indirect cost covers real income loss of family members accompanying a patient coming to the hospitals, and for informal care. The human capital method was used for the indirect cost estimation [4]. Although the friction cost method can produce more accurate results[5], we have limited availability of necessary data in Thailand. This research covered complications but not co-morbidities [1].

**Statistical and sensitivity analysis**

Stepwise multiple regression analysis [6] was employed to analyze the relationship between the cost (dependent variable) and potential predictor variables (independent variables). In the forward stepwise regression model, independent variables with a probability value of *F* statistics < 0.05 on the analysis were entered, and those with > 0.10 were removed. The statistical assumptions and models were examined for the following: normal distribution, homoscedasticity, multicollinearity, influential observations, and outliers [6]. To analyze robustness of the results due to sample data, univariate sensitivity analysis was employed [7]. Variation of the types of blood and chelation drugs was included. Simulated scenarios were the nucleic acid tested (NAT) blood use instead of the filtered blood, and the oral form of iron chelation drug use instead of the injection form (at the time of study the oral form was not registered.)

Results

**Sensitivity analysis**

There are two types of blood: namely, filtered blood and nucleic acid tested (NAT) blood. If filtered blood was substituted by NAT blood (unit prices are shown in Table III), then the average direct medical cost and total cost would increase by 6% and 4%, respectively (Table S1). In the case of chelation drugs, the injection form available in Thailand is desferrioxamine (Desferal®). Recently, oral-form deferasirox (Exjade®) has been marketed (unit prices are shown in Table III). If the injection form was substituted by the oral form (Desferal® 20 mg/kg/day for 5 days/week vs. Exjade® 20 mg/kg/day for 7 days/week), the average direct medical cost and total cost would increase by 257% and 152%, respectively (Table S1).

**Cost function**

Cost functions of the total cost of thalassemia were formulated to explore the predictor variables. Fourteen potential predictor variables are included in the model (Table S2). The costs as outcome variables were transformed into a natural log form to meet the assumption of normal distribution [8]. Two models were fitted to predict total medical cost and full cost. Both models were checked for assumptions and model diagnostics, and no violation was found [6]. For total medical cost (Table S3), the fitted model can predict 39% of variability of the cost (adjusted *R^2^* = 0.393). It found that use of iron chelation and blood transfusion increase the cost (Table S3). Levels of blood transfusion can be used to estimate the cost. Patients receiving treatment at the teaching hospital (Chulalongkorn) and the military hospital (Phramongkutklao) had a higher cost than those at the public regional hospital (Saraburi). Regarding the full cost (Table S4), the fitted model has a higher power of explanation than the previous one (adjusted *R^2^* = 0.574). Cost predictors were similar to those of the total direct medical cost, except for the hospitals and the insurance schemes. The cost at the teaching hospital (Chulalongkorn) was higher than that of the public regional (Saraburi). In addition, a patient under the Civil Servant Medical Benefit Scheme had a higher cost than one under the Universal Health Coverage Scheme.

Discussion

The cost function is useful for estimating the cost of specific patient characteristics and treatment conditions. The hospital is a statistically significant predictor of the costs. Patients receiving treatment from the teaching hospital incurred a higher cost than those in the non-teaching public hospital. The military hospital also serves as a teaching hospital for the army medical college. It has a positive effect on the cost, similar to that of the teaching hospital mentioned earlier. This phenomenon seems to be common in Thailand [9]. It is essential to note that the variation of cost among organizations is based on price variances (prices of resources used), efficiency variances (resource use behavior), and volume variances (output production) [10]. The patients under the Civil Servant Medical Benefit Scheme (CSMBS) incur a higher cost than those under the Universal Health Coverage Scheme (UC). This might be an effect of the health financing methods. Public hospitals in Thailand receive a fixed per-capita budget per person registered to the UC. Physicians are urged to control expenditures with such patients. Meanwhile, physicians are reimbursed for treatment of patients under the CSMBS on a fee-for-service basis. Therefore, the physicians do not feel any pressure to control the treatment cost. This phenomenon has been demonstrated in other studies [9, 11-13]. Similarly, the effect of insurance schemes on drug costs has been published in the United States [14]. For level of blood transfusion and iron chelation drug use, they directly indicate quantity of resource consumption. We included this in the model in order to estimate the costs of different treatment regimens. In case of a complication, it does not significantly affect the cost. Then again, this might be an effect of the sample size. We found that only 9% had complications. By disease type, average costs of ß-thal/Hb E and homozygous ß-thal were US$902 and US$1,434, respectively. However, when other variables were controlled, there was no statistically significant difference between the disease types in the cost model. The number of ß-thal/Hb E and homozygous ß-thal patients were 183 cases and 18 cases, respectively. Due to this huge difference in sample size, the effect of the disease type on the cost was inconclusive. Regarding the cost analysis, it is essential to note that it should be considered together with the treatment outcome. Despite higher cost, optimal treatment may be more cost-effective over a patient’s lifetime. People who are adequately transfused and iron-chelated will have significantly better survival rates, and thereby will be able to contribute to the country’s economy.

The estimated cost of the disease has been used in policy development, as in the approval of the National Thalassemia Plan 2007-2011 [15]. In addition to diagnosis and treatment development, it covers goals to increase treatment efficiency, and investment in technology for prevention and diagnosis. The plan also covers the development of efficient treatment and patient care regimens, and screening and diagnosis methods. These are health technology developments. Therefore, they must be evaluated as part of a health technology assessment [16]. The presentation made in this study covers categories of resources, unit costs of major resources, quantity of resources consumed, level capacity utilization (e.g., bed occupancy rate), and composition of costing results. This follows the World Health Organization guidelines [17]. The costs are classified by thalassemia type, severity, complications, blood transfusion, use of iron chelation drug, and level of ferritin. This information will be useful in future models of cost-effectiveness analysis [18]. Similarly, we can explore the economic outcomes of various treatment regimens, such as transfusion level and splenectomy.

Regarding the care of chronic illness, the National Health Security Office (NHSO) has introduced the concept of disease management [19]. The NHSO is responsible for the Universal Health Coverage Scheme (UC), which is a major public health insurance plan in Thailand. For health care financing, hospitals providing services for patients under this scheme generally receive reimbursement on a capitation basis. In contrast, the disease management program provides payment for hospitals employing capitation per illness. The inclusion of managing thalassemia is in the process of being considered under the disease management program. The detailed costing results can be used as information for a budget impact analysis by the NHSO [20, 21].

**References**
