# Supplementary material for: Economic burden of beta-thalassemia/Hb E and beta-thalassemia major in Thai children
Source: BMC Res Notes. 2010 Jan 30;3:29. doi: 10.1186/1756-0500-3-29 (PMC2835719; doi:10.1186/1756-0500-3-29)
Supplement: Additional file 2 — Table S1-S4. Results of sensitivity analysis and cost function. [file 1756-0500-3-29-S2.DOC]

**Additional file 2: Table S1-S4 (Results of sensitivity analysis and cost function)**

**Table S1: Effect of blood type and iron chelation drugs on costs**

| Category | Base case | | NAT blood | | | | Oral iron chelation | | | |
| --- | --- | --- | --- | --- | --- | --- | --- | --- | --- | --- |
|  | DMC^1^ | Total cost | DMC^1^ | % change | Total cost | % change | DMC^1^ | % change | Total cost | % change |
| Hospital |  |  |  |  |  |  |  |  |  |  |
| Saraburi (N=72) | 224.90 | 380.09 | 240.50 | 6.94 | 395.69 | 4.10 | 337.33 | 49.99 | 492.52 | 29.58 |
| Phramongkutklao (N=42) | 782.70 | 1206.23 | 826.24 | 5.56 | 1249.77 | 3.61 | 3861.72 | 393.39 | 4285.25 | 255.26 |
| Chulalongkorn (N=87) | 736.19 | 1297.38 | 788.37 | 7.09 | 1349.56 | 4.02 | 2498.35 | 239.36 | 3059.54 | 135.83 |
| Disease type |  |  |  |  |  |  |  |  |  |  |
| Beta-thal/Hb E (N=183) | 531.82 | 902.10 | 567.27 | 6.67 | 937.54 | 3.93 | 1829.28 | 243.96 | 2199.55 | 143.83 |
| Homozygous beta-thal (N=18) | 877.24 | 1434.27 | 933.04 | 6.36 | 1490.07 | 3.89 | 3837.66 | 337.47 | 4394.69 | 206.41 |
| Blood transfusion |  |  |  |  |  |  |  |  |  |  |
| No transfusion (N=41) | 93.01 | 232.87 | 96.49 | 3.74 | 236.34 | 1.49 | 180.14 | 93.67 | 319.99 | 37.42 |
| Occasional (N=40) | 239.79 | 503.03 | 255.49 | 6.55 | 518.73 | 3.12 | 465.10 | 93.96 | 728.34 | 44.79 |
| Low (N=79) | 642.22 | 1111.56 | 689.76 | 7.40 | 1159.10 | 4.28 | 2199.52 | 242.49 | 2668.86 | 140.10 |
| High (N=38) | 1104.50 | 1670.01 | 1174.22 | 6.31 | 1739.72 | 4.18 | 4577.49 | 314.44 | 5143.00 | 207.96 |
| Severity |  |  |  |  |  |  |  |  |  |  |
| Severe (N=94) | 674.95 | 1023.01 | 720.02 | 6.68 | 1068.07 | 4.41 | 2596.31 | 284.67 | 2944.36 | 187.82 |
| Nonsevere (N=106) | 465.15 | 888.77 | 495.86 | 6.60 | 919.48 | 3.46 | 1503.97 | 223.33 | 1927.59 | 116.88 |
| Ferritin level |  |  |  |  |  |  |  |  |  |  |
| < 2,500 ng/ml (N=77) | 644.81 | 1146.20 | 692.66 | 7.42 | 1194.05 | 4.17 | 2328.53 | 261.12 | 2829.92 | 146.90 |
| > 2,500 ng/ml (N=50) | 916.78 | 1396.69 | 972.41 | 6.07 | 1452.32 | 3.98 | 4063.56 | 343.24 | 4543.47 | 225.30 |
| Complications |  |  |  |  |  |  |  |  |  |  |
| Yes (N=18) | 942.43 | 1385.84 | 990.59 | 5.11 | 1433.99 | 3.47 | 4660.25 | 394.49 | 5103.66 | 268.27 |
| No (N=183) | 525.41 | 906.86 | 561.61 | 6.89 | 943.06 | 3.99 | 1748.37 | 232.76 | 2129.82 | 134.86 |
| Total |  |  |  |  |  |  |  |  |  |  |
| Mean | 562.76 | 949.75 | 600.03 | 6.62 | 987.02 | 3.92 | 2009.14 | 257.02 | 2396.13 | 152.29 |
| Standard Deviation (SD) | 606.10 | n/a | 630.91 | 4.09 | 1050.96 | n/a | 3160.83 | 421.51 | 3393.65 | n/a |
| Median | 362.66 | 654.19 | 395.17 | 8.97 | 687.01 | 5.02 | 389.75 | 7.47 | 708.30 | 8.27 |
| 95%CI |  |  |  |  |  |  |  |  |  |  |
| Lower | 478.46 | 806.48 | 512.28 | 7.07 | 840.85 | 4.26 | 1569.51 | 228.04 | 1924.12 | 138.58 |
| Upper | 647.06 | 1093.03 | 687.78 | 6.29 | 1133.20 | 3.68 | 2448.77 | 278.45 | 2868.14 | 162.40 |

^1^ = Total direct medical cost

**Table S2: Variables included in the model**

| Ln of total direct medical cost; Natural log form of total direct medical cost (US$) |
| --- |
| Ln of full cost; Natural log form of the full cost (US$) |
| Age; years |
| Hospital-PK; Phramongkutklao Hospital vs. Saraburi Hospital (reference) |
| Hospital-CL; Chulalongkorn Hospital vs. Saraburi Hospital (reference) |
| Non HbH; Homozygous ß vs. ß thalassemia/Hb E (reference) |
| Severity; severe vs. non-severe (reference) |
| Splenectomy; yes vs. no (reference) |
| Complications; yes vs. no (reference) |
| Desferal use; yes vs. no (reference) |
| Low blood transfusion ; yes vs. no transfusion (reference) |
| Occasional blood transfusion; yes vs. no transfusion (reference) |
| High blood transfusion; yes vs. no transfusion (reference) |
| Social Security Scheme*; yes vs. Universal Health Coverage** (reference) |
| Civil Servant Medical Benefit Scheme; yes vs. Universal Health Coverage (reference) |
| Out-of-Pocket; yes vs. Universal Health Coverage (reference) |

* Social Security Scheme is an insurance scheme for private workers.

** Universal health coverage is a basic insurance scheme for people who are not under the Social Security Scheme or the Civil Servant Medical Benefit Scheme.

**Table S3: Fitted explanatory model of the total medical cost**

|  | Unstandardized coefficients | | *t* | Sig. | 95% confidence interval for B | |
| --- | --- | --- | --- | --- | --- | --- |
|  | *B* | Std. Error |  |  | Lower Bound | Upper Bound |
| (Constant) | 3.018 | 0.163 | 18.478 | 0.000 | 2.696 | 3.341 |
| Desferal use | 0.306 | 0.179 | 1.716 | 0.088 | -0.046 | 0.659 |
| Hospital-CL | 0.859 | 0.177 | 4.844 | 0.000 | 0.509 | 1.209 |
| Hospital-PK | 0.639 | 0.218 | 2.935 | 0.004 | 0.209 | 1.068 |
| High blood transfusion | 1.315 | 0.243 | 5.421 | 0.000 | 0.836 | 1.793 |
| Occasional blood transfusion | 0.992 | 0.191 | 5.186 | 0.000 | 0.614 | 1.369 |
| Low blood transfusion | 0.630 | 0.212 | 2.975 | 0.003 | 0.212 | 1.048 |

Adjusted *R^2^* = 0.393, *p* = 0.000

**Table S4:** **Fitted explanatory model of full cost**

|  | Unstandardized coefficients | | *t* | Sig. | 95% confidence interval for B | |
| --- | --- | --- | --- | --- | --- | --- |
|  | *B* | Std. Error |  |  | Lower Bound | Upper Bound |
| (Constant) | 4.848 | 0.123 |  | 39.262 | 0.000 | 4.605 |
| Desferal use | 0.811 | 0.135 | 0.336 | 6.004 | 0.000 | 0.545 |
| Occasional blood transfusion | 1.331 | 0.154 | 0.553 | 8.668 | 0.000 | 1.029 |
| High blood transfusion | 1.542 | 0.195 | 0.514 | 7.925 | 0.000 | 1.158 |
| Low blood transfusion | 0.732 | 0.169 | 0.249 | 4.322 | 0.000 | 0.398 |
| Hospital-CL | 0.292 | 0.116 | 0.123 | 2.516 | 0.013 | 0.063 |
| Civil Servant Medical Benefit Scheme | 0.392 | 0.173 | 0.106 | 2.266 | 0.025 | 0.051 |

Adjusted *R^2^* = 0.574, *p* = 0.000
